# Supplementary material for: Comparisons of Hematological and Biochemical Profiles in Brahman and Yunling Cattle
Source: Animals (Basel). 2022 Jul 15;12(14):1813. doi: 10.3390/ani12141813 (PMC9311853; doi:10.3390/ani12141813)
Supplement: Supplementary file 1 [file animals-12-01813-s001.zip › animals-1738787-supplementary.pdf]

Supplementary Figure S1: The distribution of variables for normality with histograms, for all data of hematological and biochemical of Brahman and Yunling Cattle.

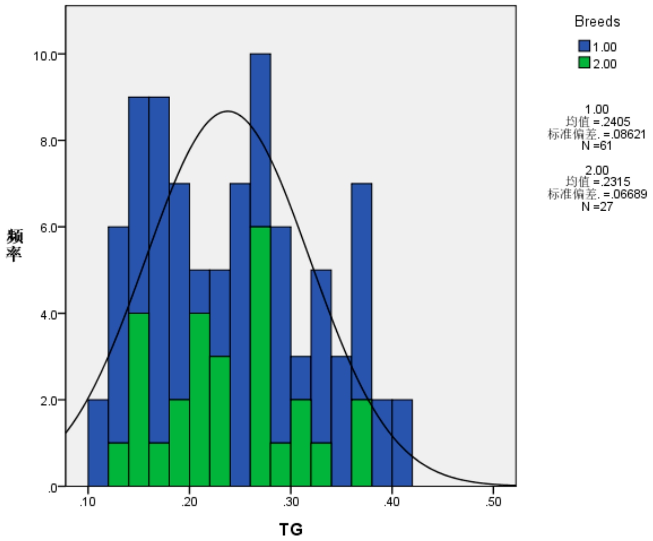

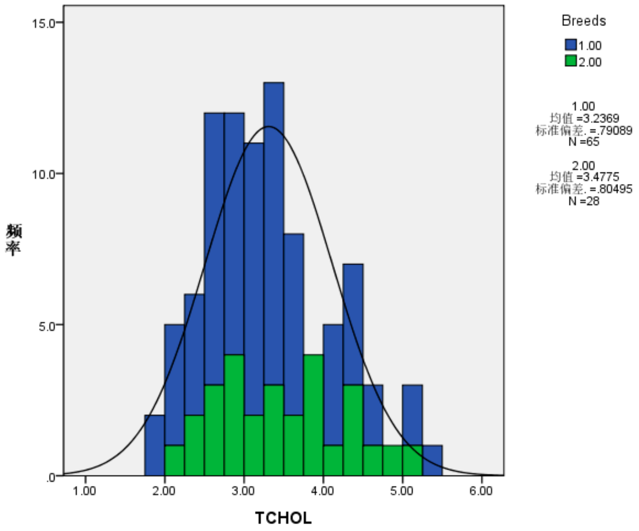

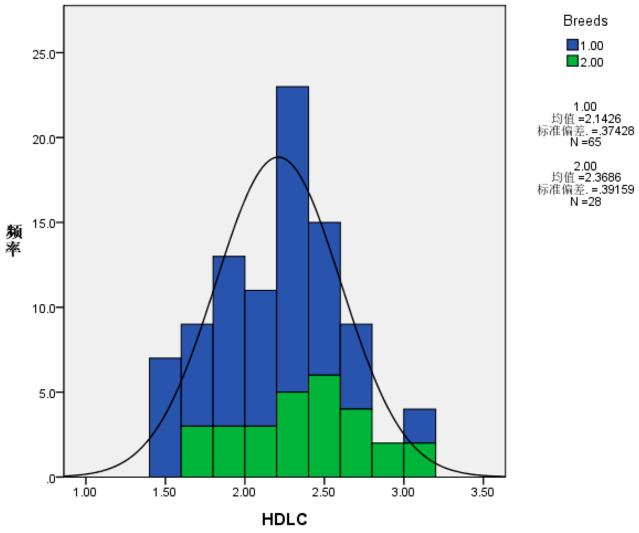

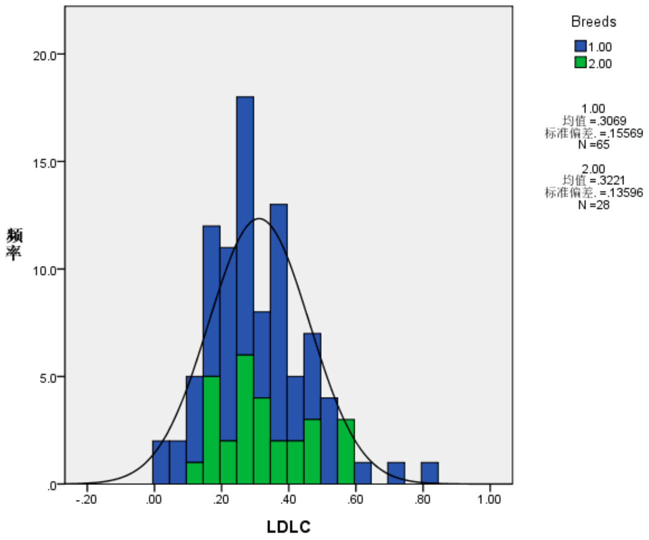
Breeds
1.00
2.00
1.00
均值 =.3069
标准偏差. =.15569
N =65
2.00
均值 =.3221
标准偏差. =.13596
N =28
频率
20.0
15.0
10.0
5.0
.0
-.20
.00
.20
.40
.60
.80
1.00
LDLC

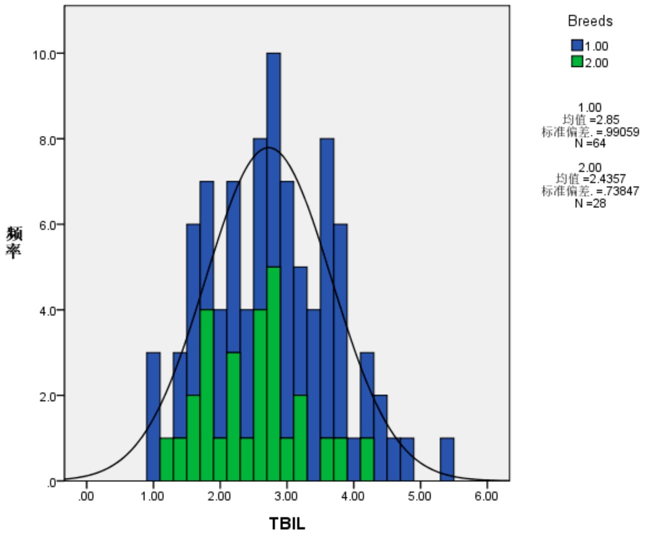
Breeds
1.00
2.00
1.00
均值 =2.85
标准偏差. =.99059
N =64
2.00
均值 =2.4357
标准偏差. =.73847
N =28
频率
10.0
8.0
6.0
4.0
2.0
.0
.00
1.00
2.00
3.00
4.00
5.00
6.00
TBIL

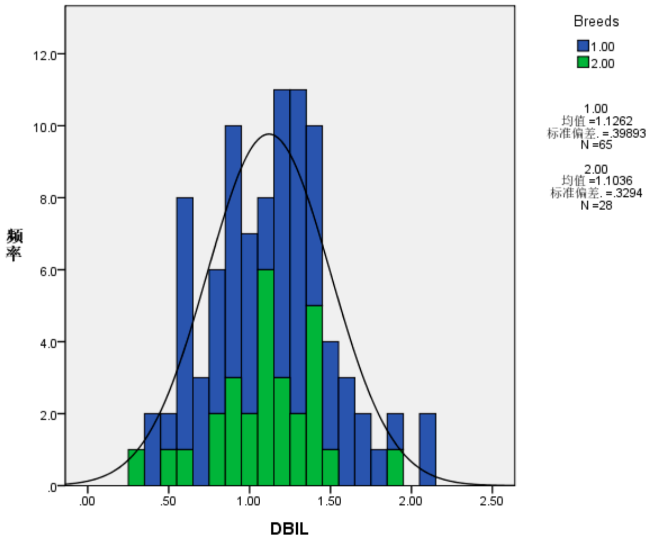
Breeds
1.00
2.00
1.00
均值 =1.1262
标准偏差. =.39893
N =65
2.00
均值 =1.1036
标准偏差. =.3294
N =28
频率
12.0
10.0
8.0
6.0
4.0
2.0
.0
.00
.50
1.00
1.50
2.00
2.50
DBIL

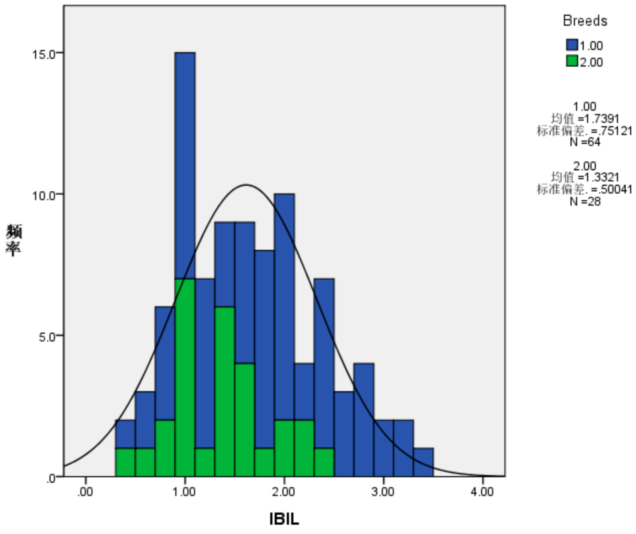
频率
IBIL
Breeds
1.00
2.00
1.00
均值 =1.7391
标准偏差. =.75121
N =64
2.00
均值 =1.3321
标准偏差. =.50041
N =28

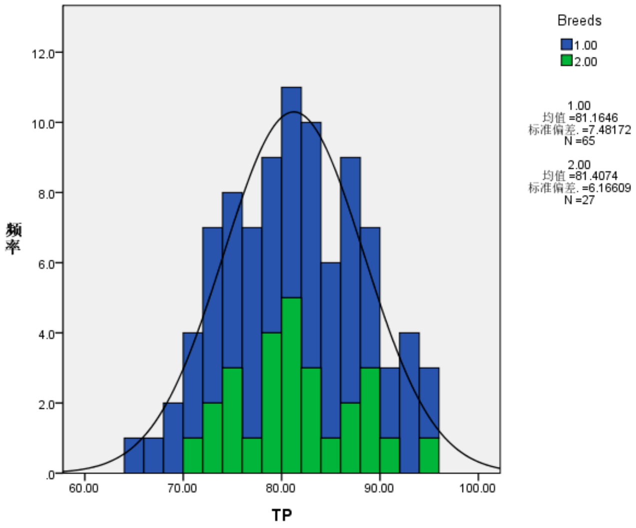
频率
TP
Breeds
1.00
2.00
1.00
均值 =81.1646
标准偏差. =7.48172
N =65
2.00
均值 =81.4074
标准偏差. =6.16609
N =27

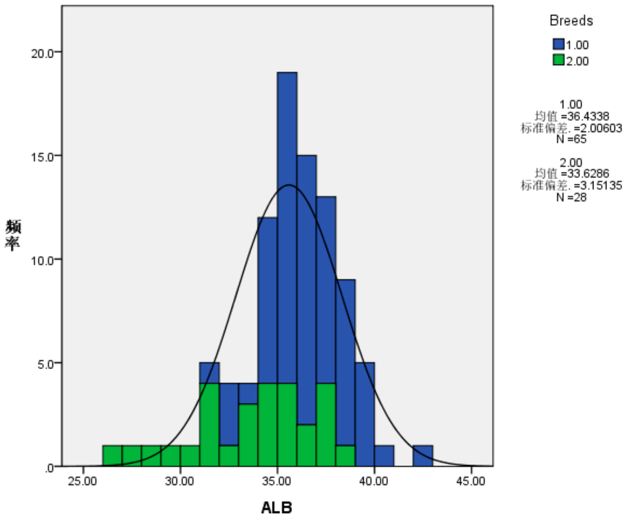
频率
ALB
Breeds
1.00
2.00
1.00
均值 =36.4338
标准偏差. =2.00603
N =65
2.00
均值 =33.6286
标准偏差. =3.15135
N =28

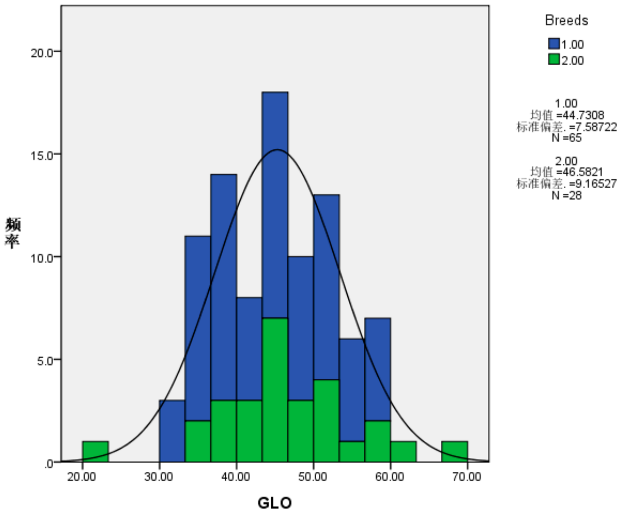
频率
GLO
Breeds
1.00
2.00
1.00
均值 =44.7308
标准偏差. =7.58722
N =65
2.00
均值 =46.5821
标准偏差. =9.16527
N =28

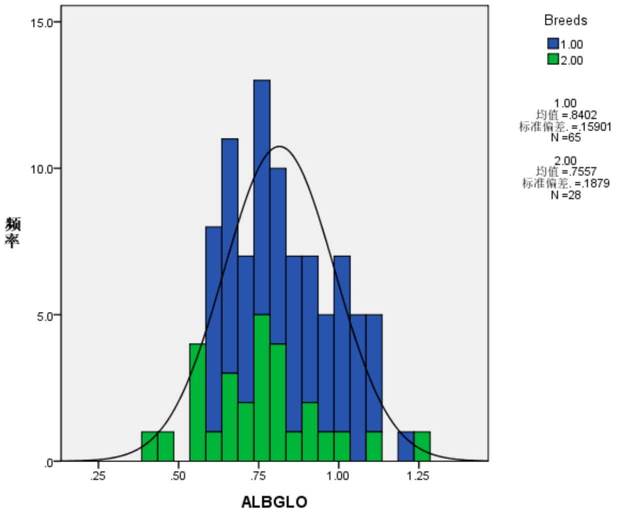
频率
ALBGLO
Breeds
1.00
2.00
1.00
均值 =.8402
标准偏差. =.15901
N =65
2.00
均值 =.7557
标准偏差. =.1879
N =28

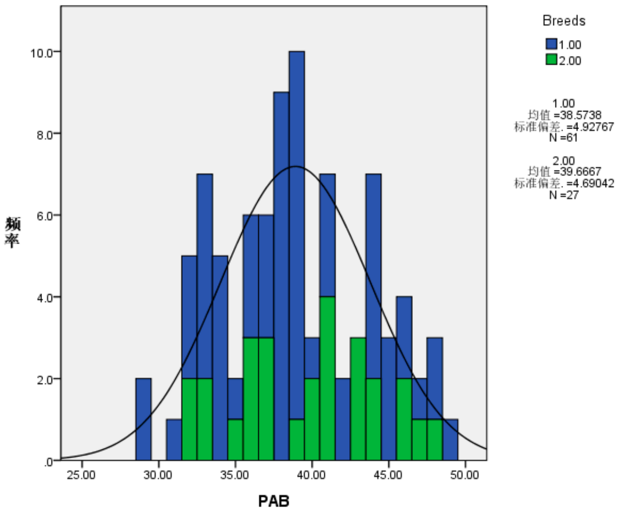
频率
PAB
Breeds
1.00
2.00
1.00
均值 =38.5738
标准偏差. =4.92767
N =61
2.00
均值 =39.6667
标准偏差. =4.69042
N =27

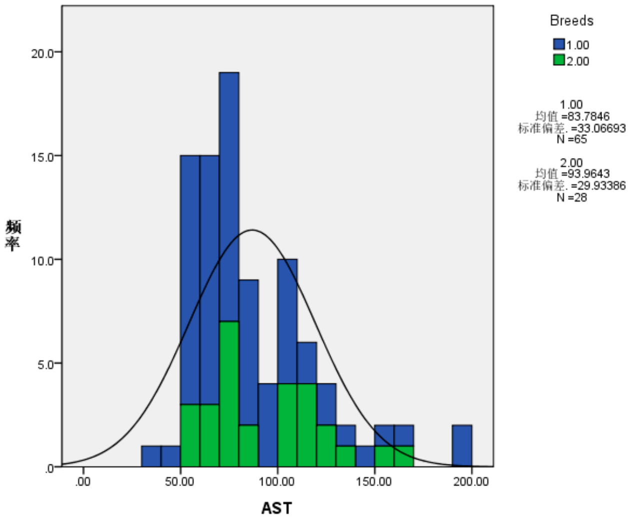
20.0
15.0
10.0
5.0
0
频率
.00
50.00
100.00
150.00
200.00
AST
Breeds
1.00
2.00
1.00
均值 =83.7846
标准偏差. =33.06693
N =65
2.00
均值 =93.9643
标准偏差. =29.93386
N =28

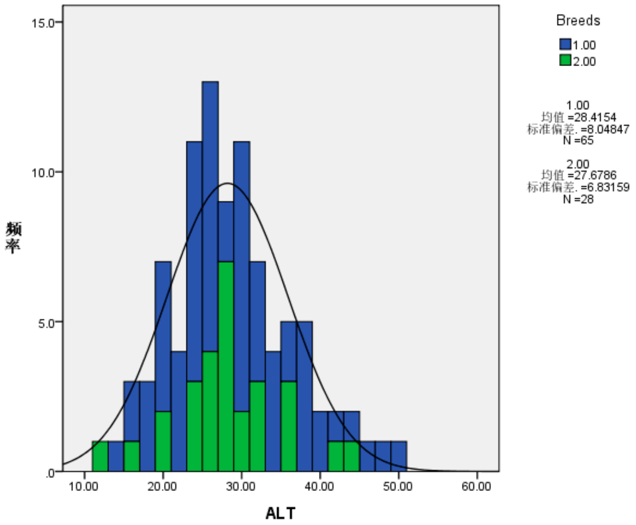
15.0
10.0
5.0
0
频率
10.00
20.00
30.00
40.00
50.00
60.00
ALT
Breeds
1.00
2.00
1.00
均值 =28.4154
标准偏差. =8.04847
N =65
2.00
均值 =27.6786
标准偏差. =6.83159
N =28

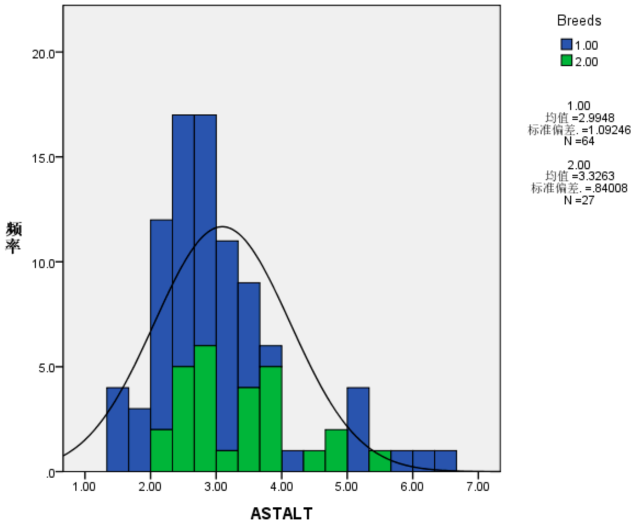
20.0
15.0
10.0
5.0
.0
频率
1.00
2.00
3.00
4.00
5.00
6.00
7.00
ASTALT
Breeds
1.00
2.00
1.00
均值 =2.9948
标准偏差. =1.09246
N =64
2.00
均值 =3.3263
标准偏差. =.84008
N =27

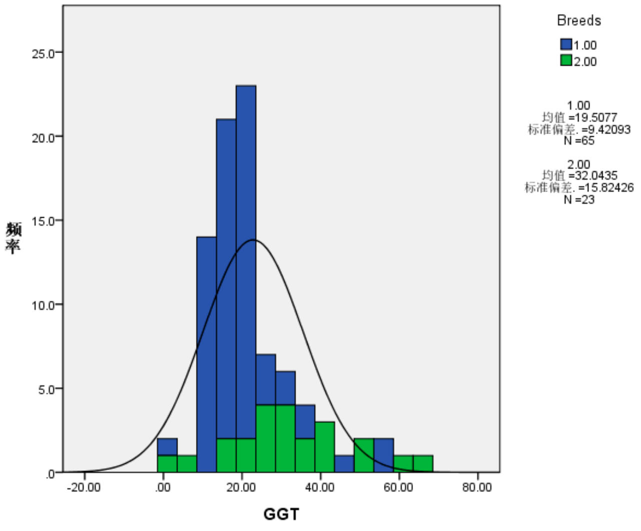

25.0
20.0
15.0
10.0
5.0
.0
频率
-20.00
.00
20.00
40.00
60.00
80.00
GGT
Breeds
1.00
2.00
1.00
均值 =19.5077
标准偏差. =9.42093
N =65
2.00
均值 =32.0435
标准偏差. =15.82426
N =23

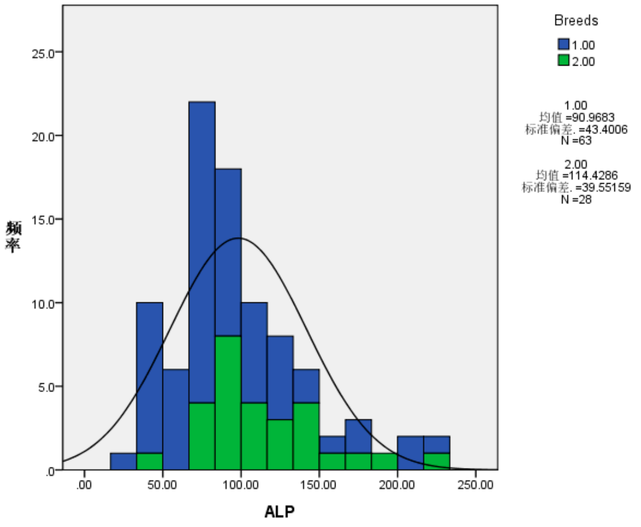

25.0
20.0
15.0
10.0
5.0
.0
频率
.00
50.00
100.00
150.00
200.00
250.00
ALP
Breeds
1.00
2.00
1.00
均值 =90.9683
标准偏差. =43.4006
N =63
2.00
均值 =114.4286
标准偏差. =39.55159
N =28

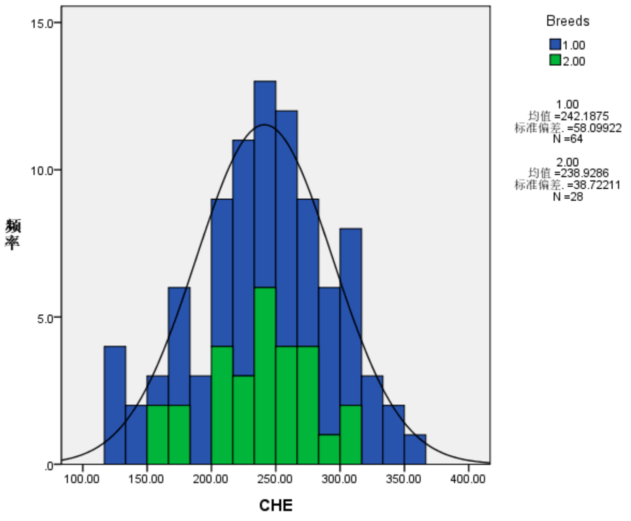

15.0
10.0
5.0
.0
频率
100.00
150.00
200.00
250.00
300.00
350.00
400.00
CHE
Breeds
1.00
2.00
1.00
均值 =242.1875
标准偏差. =58.09922
N =64
2.00
均值 =238.9286
标准偏差. =38.72211
N =28

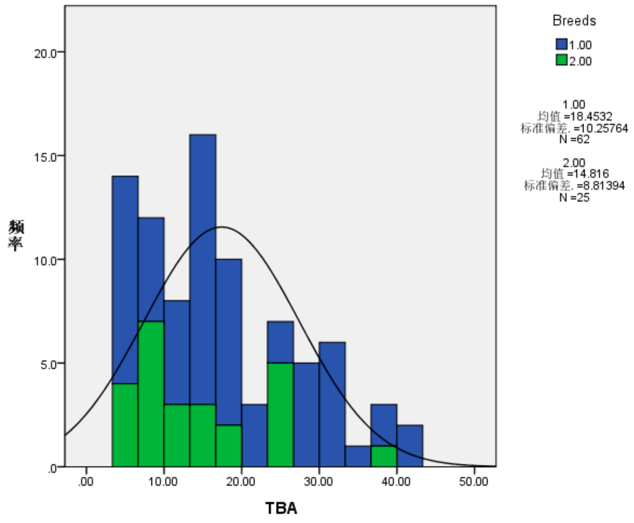
频率
20.0
15.0
10.0
5.0
.0
.00
10.00
20.00
30.00
40.00
50.00
TBA
Breeds
1.00
2.00
1.00
均值 =18.4532
标准偏差. =10.25764
N =62
2.00
均值 =14.816
标准偏差. =8.81394
N =25

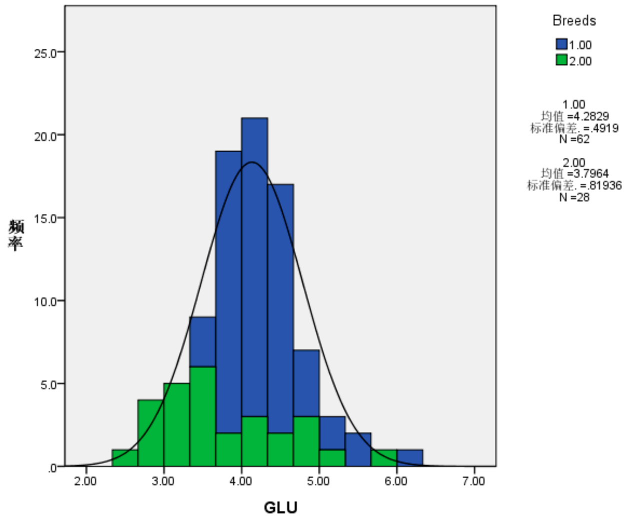
频率
25.0
20.0
15.0
10.0
5.0
.0
2.00
3.00
4.00
5.00
6.00
7.00
GLU
Breeds
1.00
2.00
1.00
均值 =4.2829
标准偏差. =.4919
N =62
2.00
均值 =3.7964
标准偏差. =.81936
N =28

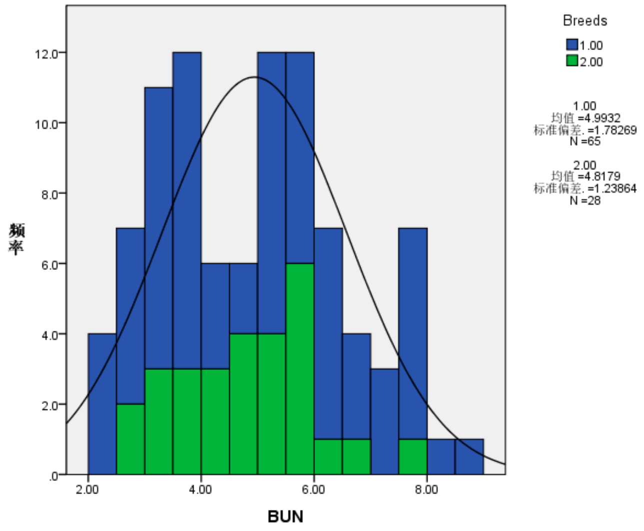
频率
12.0
10.0
8.0
6.0
4.0
2.0
.0
2.00
4.00
6.00
8.00
BUN
Breeds
1.00
2.00
1.00
均值 =4.9932
标准偏差. =1.78269
N =65
2.00
均值 =4.8179
标准偏差. =1.23864
N =28

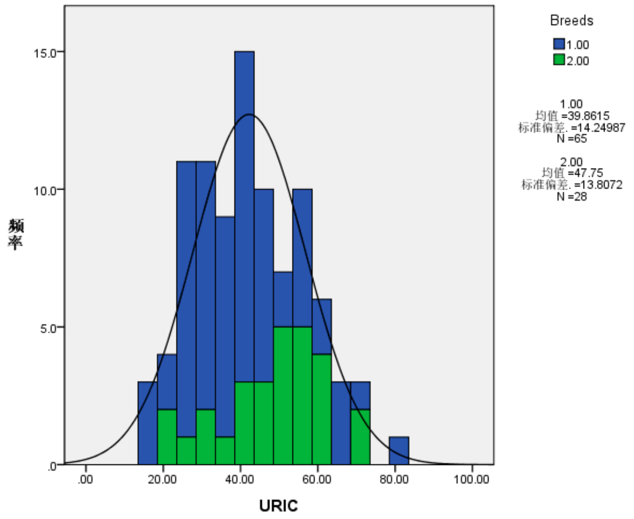
频率
URIC
Breeds
1.00
2.00
1.00
均值 =39.8615
标准偏差. =14.24987
N =65
2.00
均值 =47.75
标准偏差. =13.8072
N =28

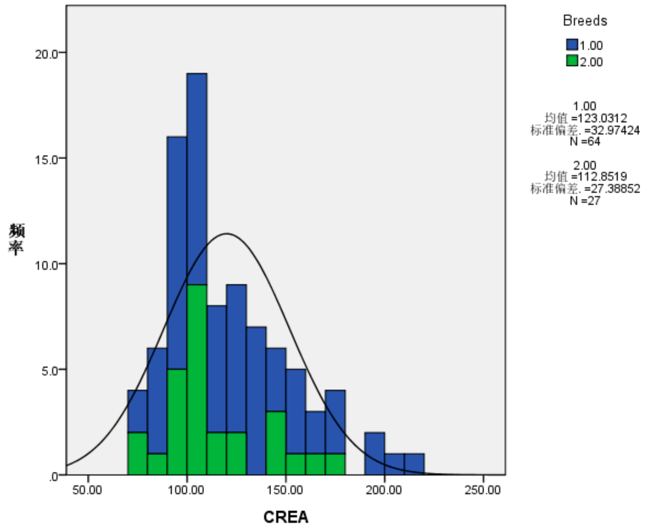
频率
CREA
Breeds
1.00
2.00
1.00
均值 =123.0312
标准偏差. =32.97424
N =64
2.00
均值 =112.8519
标准偏差. =27.38852
N =27

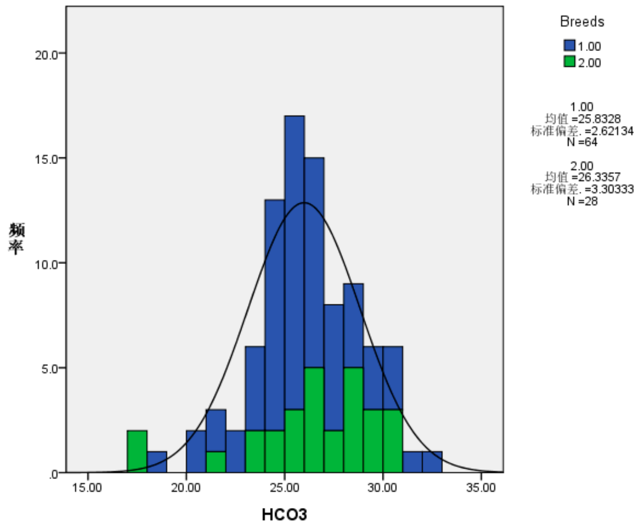
频率
HCO3
Breeds
1.00
2.00
1.00
均值 =25.8328
标准偏差. =2.62134
N =64
2.00
均值 =26.3357
标准偏差. =3.30333
N =28

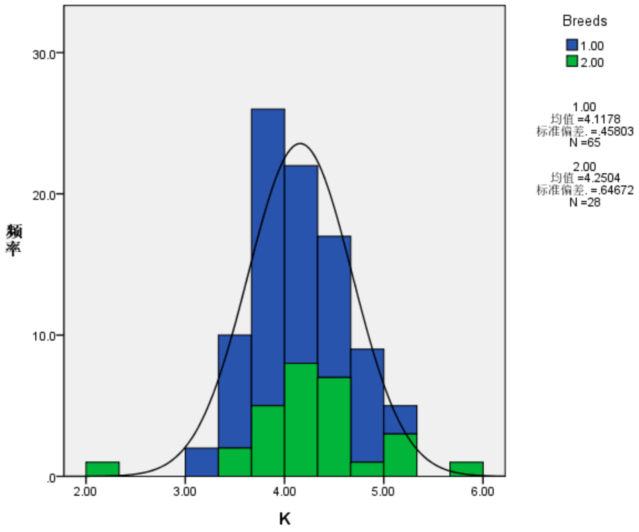
Breeds
1.00
2.00
1.00
均值 =4.1178
标准偏差. =.45803
N =65
2.00
均值 =4.2504
标准偏差. =.64672
N =28
频率
30.0
20.0
10.0
.0
2.00
3.00
4.00
5.00
6.00
K

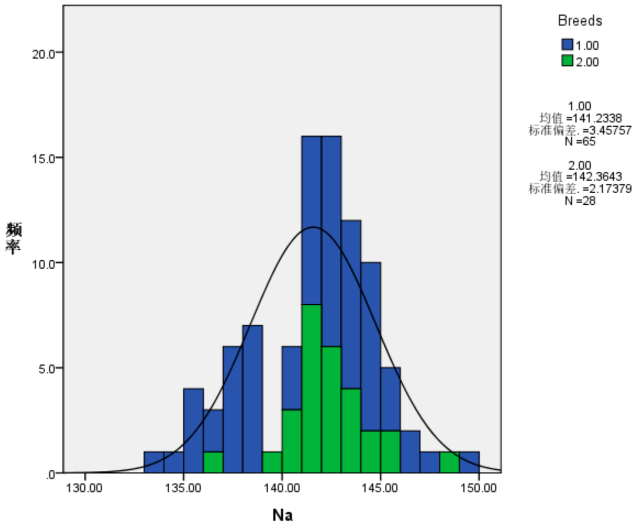
Breeds
1.00
2.00
1.00
均值 =141.2338
标准偏差. =3.45757
N =65
2.00
均值 =142.3643
标准偏差. =2.17379
N =28
频率
20.0
15.0
10.0
5.0
.0
130.00
135.00
140.00
145.00
150.00
Na

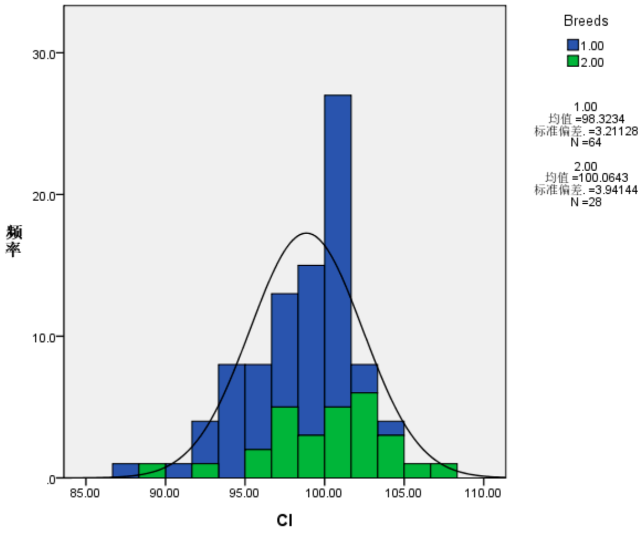
Breeds
1.00
2.00
1.00
均值 =98.3234
标准偏差. =3.21128
N =64
2.00
均值 =100.0643
标准偏差. =3.94144
N =28
频率
30.0
20.0
10.0
.0
85.00
90.00
95.00
100.00
105.00
110.00
Cl

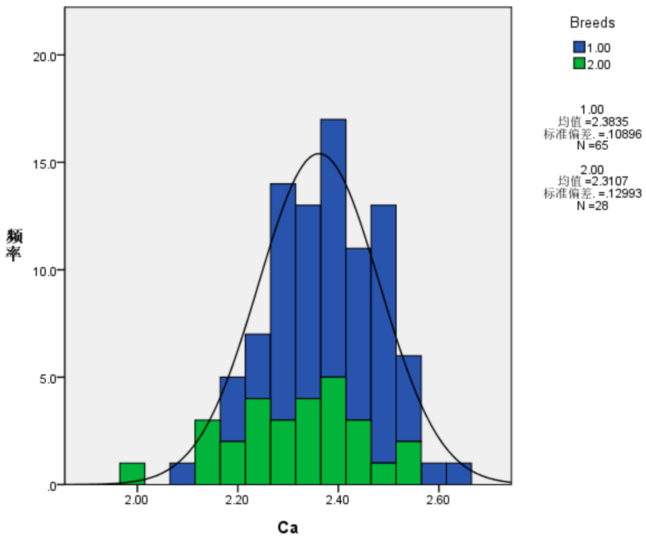
Breeds
1.00
2.00
1.00
均值 =2.3835
标准偏差. =.10896
N =65
2.00
均值 =2.3107
标准偏差. =.12993
N =28
频率
20.0
15.0
10.0
5.0
.0
2.00
2.20
2.40
2.60
Ca

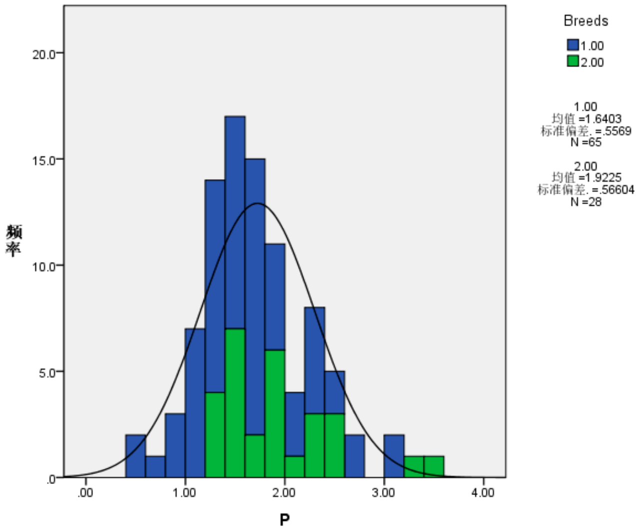
Breeds
1.00
2.00
1.00
均值 =1.6403
标准偏差. =.5569
N =65
2.00
均值 =1.9225
标准偏差. =.56604
N =28
频率
20.0
15.0
10.0
5.0
.0
.00
1.00
2.00
3.00
4.00
P

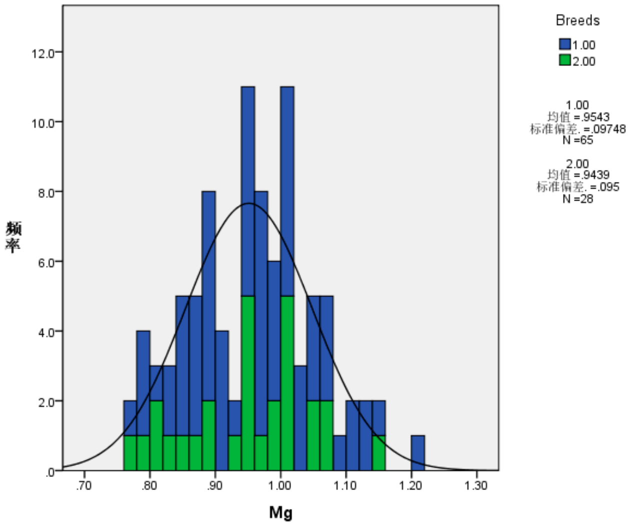
Breeds
1.00
2.00
1.00
均值 =.9543
标准偏差. =.09748
N =65
2.00
均值 =.9439
标准偏差. =.095
N =28
频率
12.0
10.0
8.0
6.0
4.0
2.0
.0
.70
.80
.90
1.00
1.10
1.20
1.30
Mg

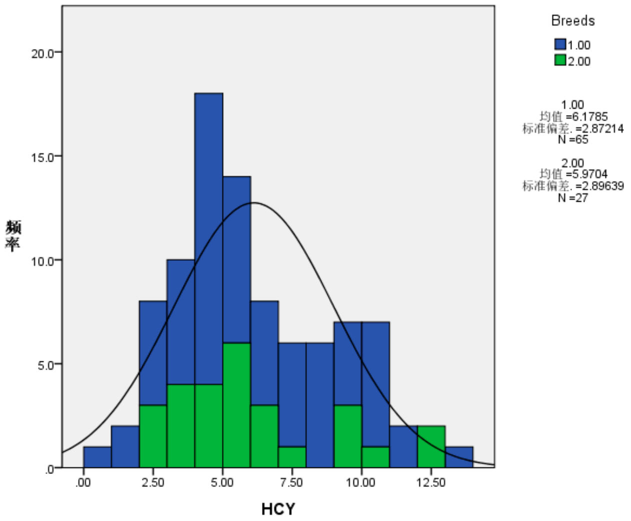

Breeds
1.00
2.00
1.00
均值 =6.1785
标准偏差. =2.87214
N =65
2.00
均值 =5.9704
标准偏差. =2.89639
N =27
频率
HCY

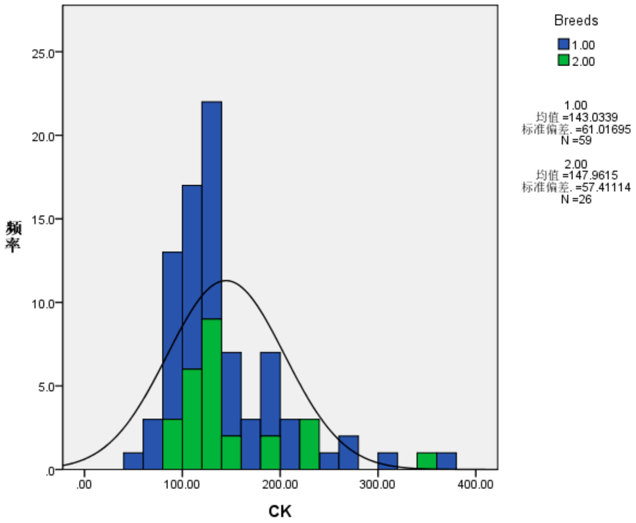

Breeds
1.00
2.00
1.00
均值 =143.0339
标准偏差. =61.01695
N =59
2.00
均值 =147.9615
标准偏差. =57.41114
N =26
频率
CK

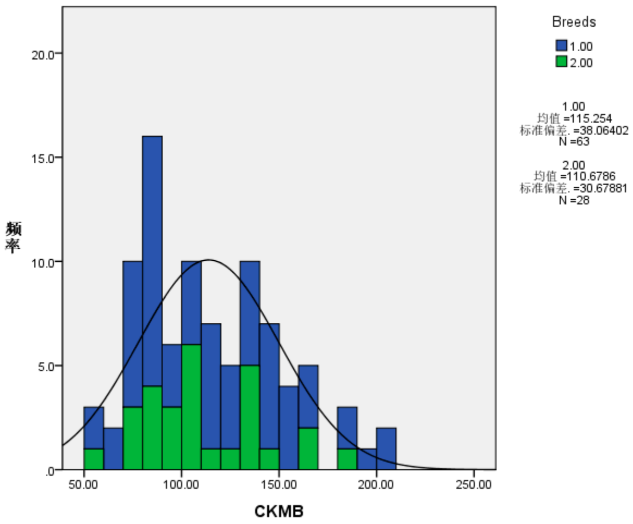

Breeds
1.00
2.00
1.00
均值 =115.254
标准偏差. =38.06402
N =63
2.00
均值 =110.6786
标准偏差. =30.67881
N =28
频率
CKMB

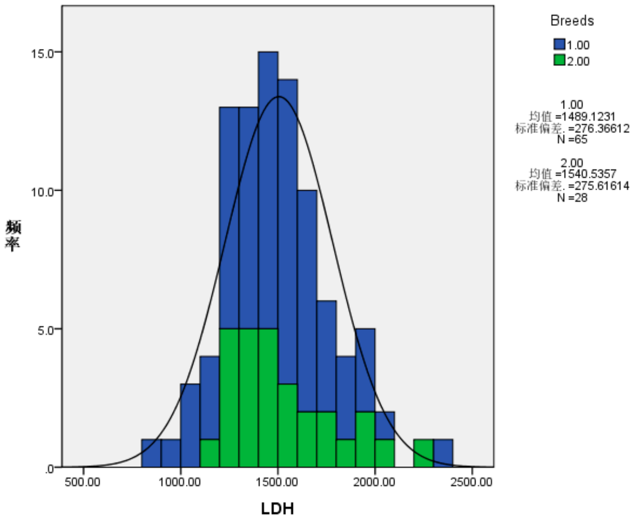
Breeds
1.00
2.00
1.00
均值 =1489.1231
标准偏差. =276.36612
N =65
2.00
均值 =1540.5357
标准偏差. =275.61614
N =28
频率
15.0
10.0
5.0
0
500.00
1000.00
1500.00
2000.00
2500.00
LDH

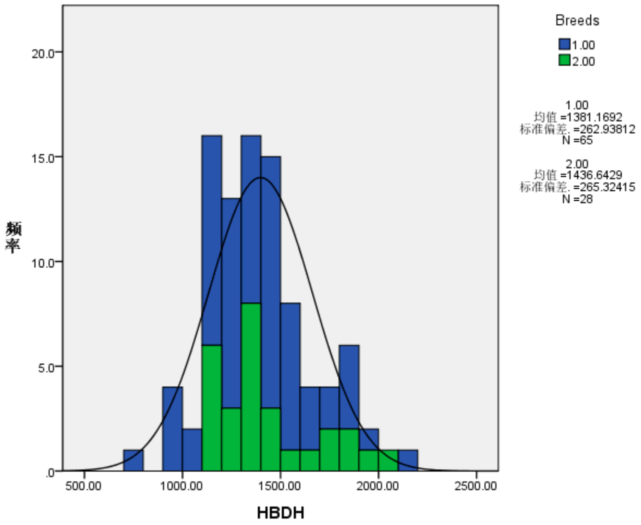
Breeds
1.00
2.00
1.00
均值 =1381.1692
标准偏差. =262.93812
N =65
2.00
均值 =1436.6429
标准偏差. =265.32415
N =28
频率
20.0
15.0
10.0
5.0
0
500.00
1000.00
1500.00
2000.00
2500.00
HBDH

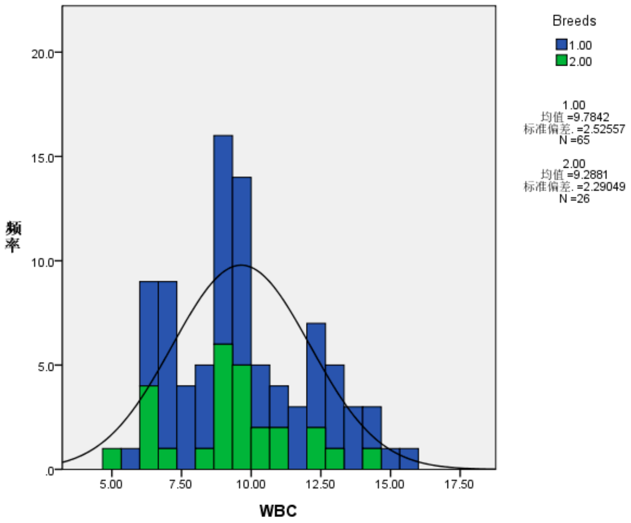
Breeds
1.00
2.00
1.00
均值 =9.7842
标准偏差. =2.52557
N =65
2.00
均值 =9.2881
标准偏差. =2.29049
N =26
频率
20.0
15.0
10.0
5.0
0
5.00
7.50
10.00
12.50
15.00
17.50
WBC

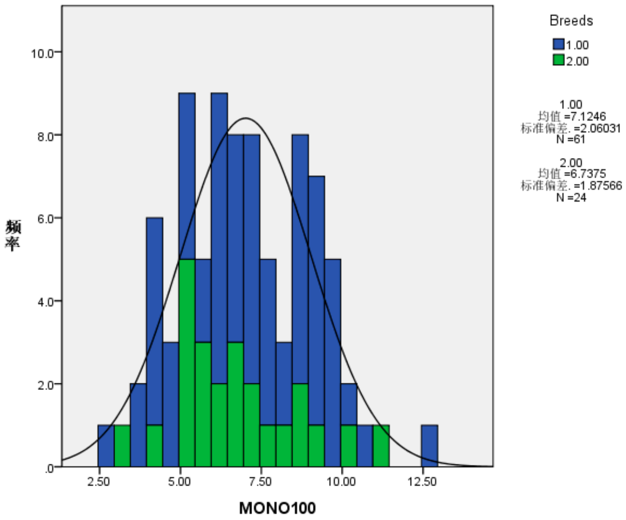

Breeds
1.00
2.00
1.00
均值 =7.1246
标准偏差. =2.06031
N =61
2.00
均值 =6.7375
标准偏差. =1.87566
N =24
频率
MONO100

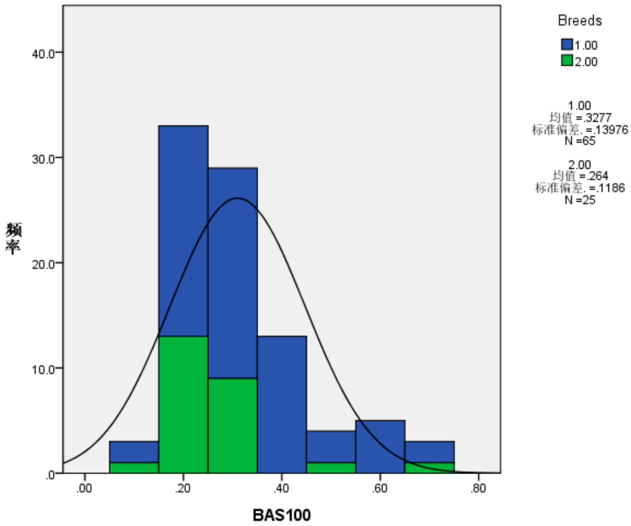

Breeds
1.00
2.00
1.00
均值 =.3277
标准偏差. =.13976
N =65
2.00
均值 =.264
标准偏差. =.1186
N =25
频率
BAS100

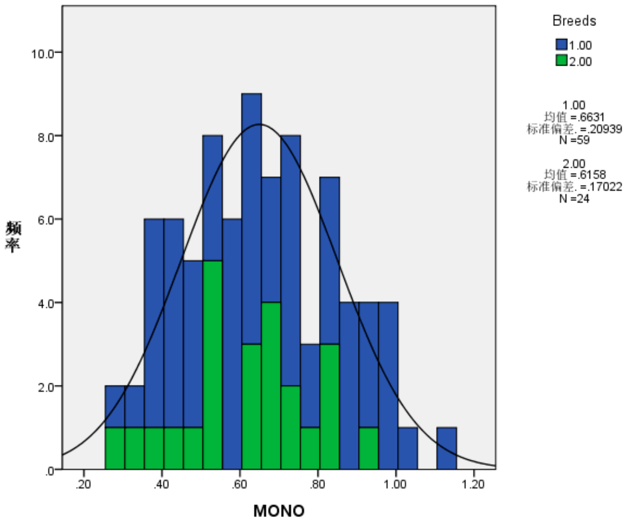

Breeds
1.00
2.00
1.00
均值 =.6631
标准偏差. =.20939
N =59
2.00
均值 =.6158
标准偏差. =.17022
N =24
频率
MONO

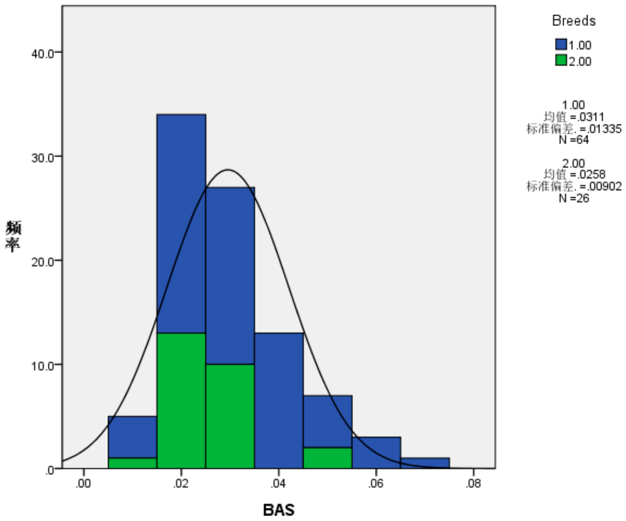
Breeds
1.00
2.00
1.00
均值 =.0311
标准偏差. =.01335
N =64
2.00
均值 =.0258
标准偏差. =.00902
N =26
频率
BAS

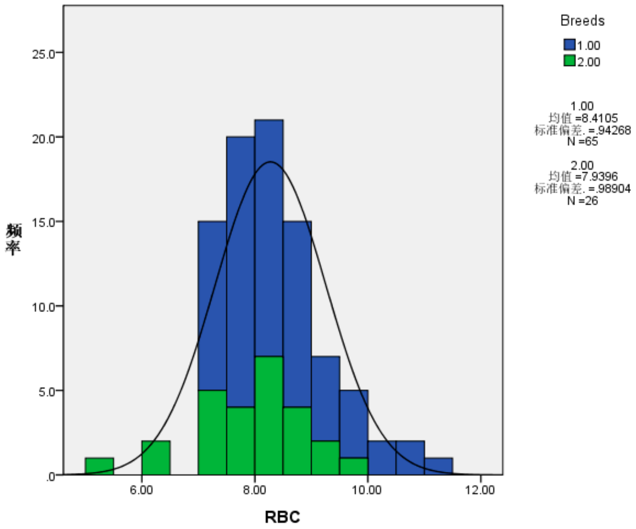
Breeds
1.00
2.00
1.00
均值 =8.4105
标准偏差. =.94268
N =65
2.00
均值 =7.9396
标准偏差. =.98904
N =26
频率
RBC

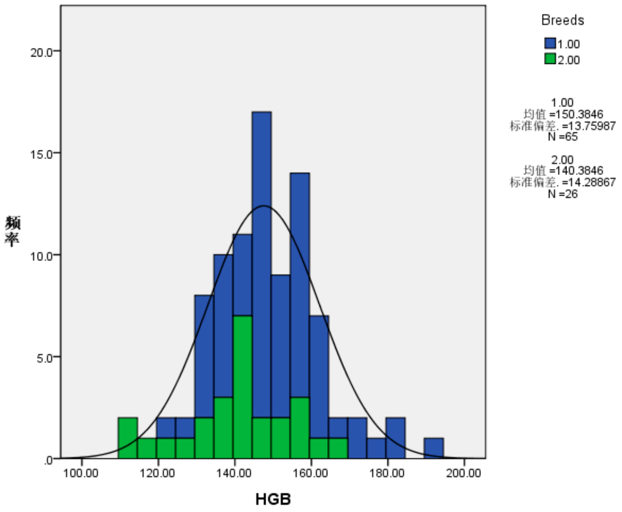
Breeds
1.00
2.00
1.00
均值 =150.3846
标准偏差. =13.75987
N =65
2.00
均值 =140.3846
标准偏差. =14.28867
N =26
频率
HGB

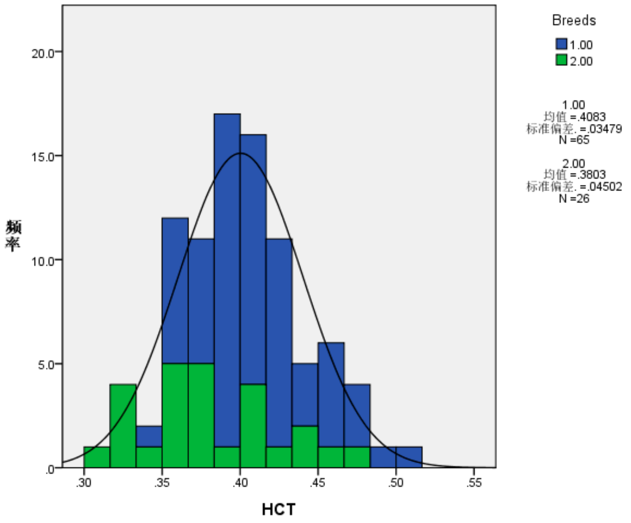
Breeds
1.00
2.00
1.00
均值 =.4083
标准偏差. =.03479
N =65
2.00
均值 =.3803
标准偏差. =.04502
N =26
频率
HCT

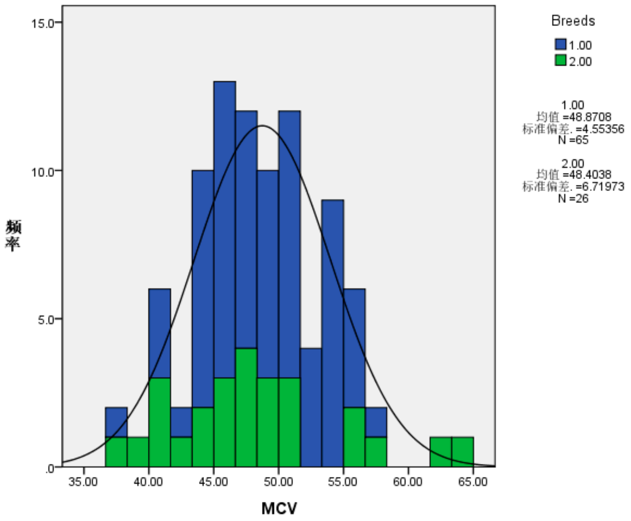
Breeds
1.00
2.00
1.00
均值 =48.8708
标准偏差. =4.55356
N =65
2.00
均值 =48.4038
标准偏差. =6.71973
N =26
频率
MCV

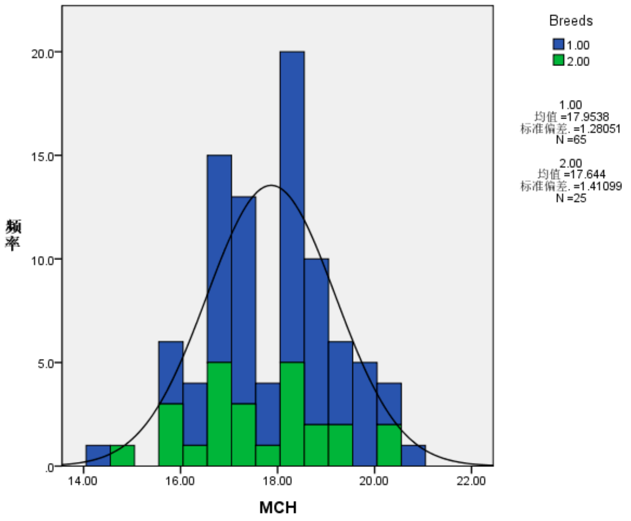
Breeds
1.00
2.00
1.00
均值 =17.9538
标准偏差. =1.28051
N =65
2.00
均值 =17.644
标准偏差. =1.41099
N =25
频率
MCH

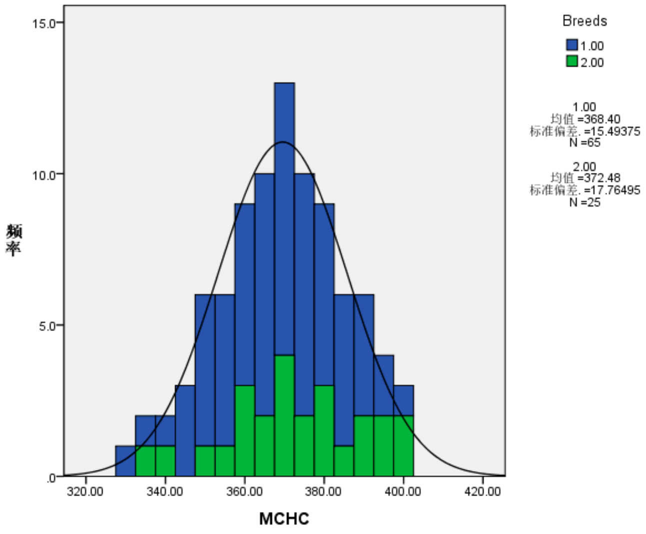

15.0
10.0
5.0
.0
频率
320.00
340.00
360.00
380.00
400.00
420.00
MCHC
Breeds
1.00
2.00
1.00
均值 =368.40
标准偏差. =15.49375
N =65
2.00
均值 =372.48
标准偏差. =17.76495
N =25

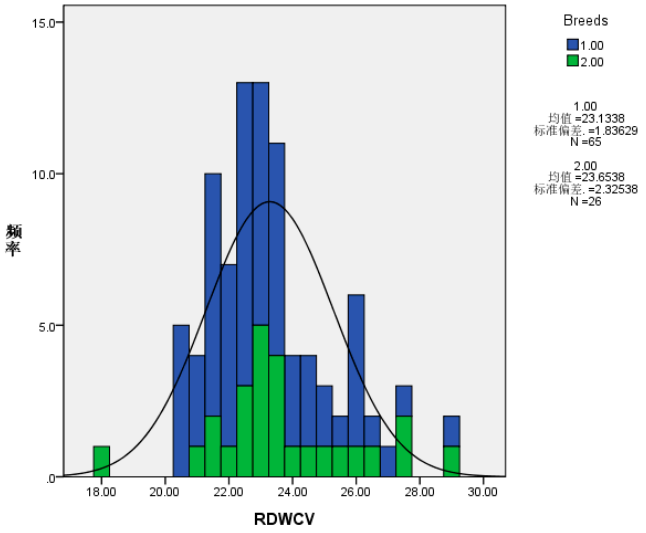

15.0
10.0
5.0
.0
频率
18.00
20.00
22.00
24.00
26.00
28.00
30.00
RDWCV
Breeds
1.00
2.00
1.00
均值 =23.1338
标准偏差. =1.83629
N =65
2.00
均值 =23.6538
标准偏差. =2.32538
N =26

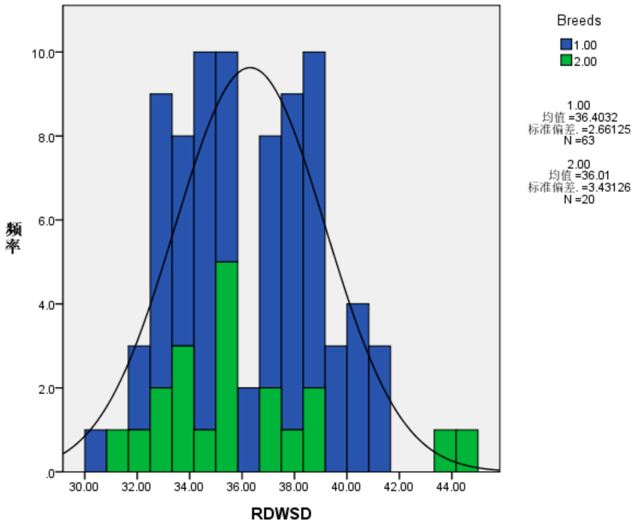

10.0
8.0
6.0
4.0
2.0
.0
频率
30.00
32.00
34.00
36.00
38.00
40.00
42.00
44.00
RDWSD
Breeds
1.00
2.00
1.00
均值 =36.4032
标准偏差. =2.66125
N =63
2.00
均值 =36.01
标准偏差. =3.43126
N =20

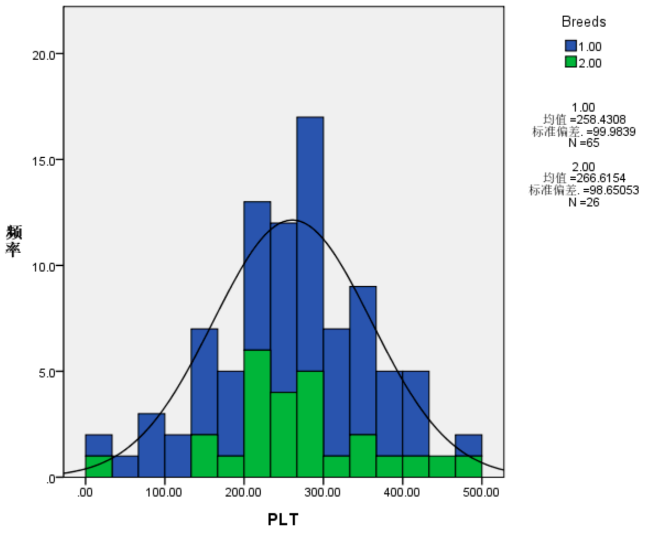
Breeds
1.00
2.00
1.00
均值 =258.4308
标准偏差. =99.9839
N =65
2.00
均值 =266.6154
标准偏差. =98.65053
N =26
频率
PLT

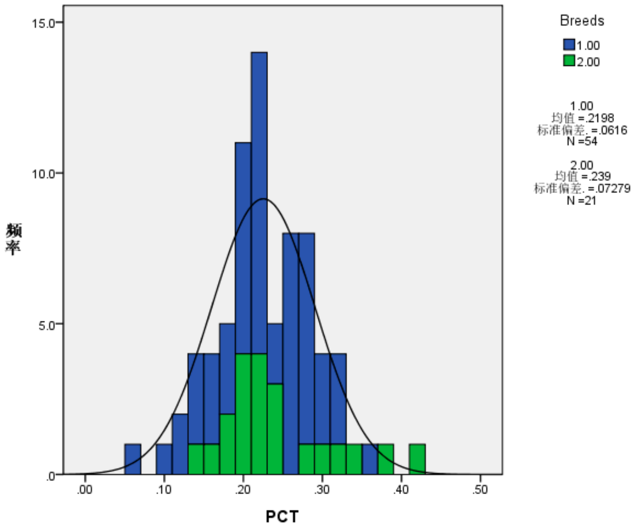
Breeds
1.00
2.00
1.00
均值 =.2198
标准偏差. =.0616
N =54
2.00
均值 =.239
标准偏差. =.07279
N =21
频率
PCT

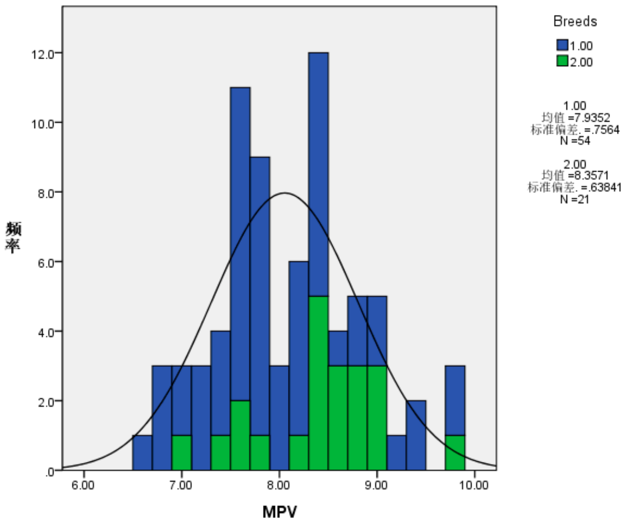
Breeds
1.00
2.00
1.00
均值 =7.9352
标准偏差. =.7564
N =54
2.00
均值 =8.3571
标准偏差. =.63841
N =21
频率
MPV

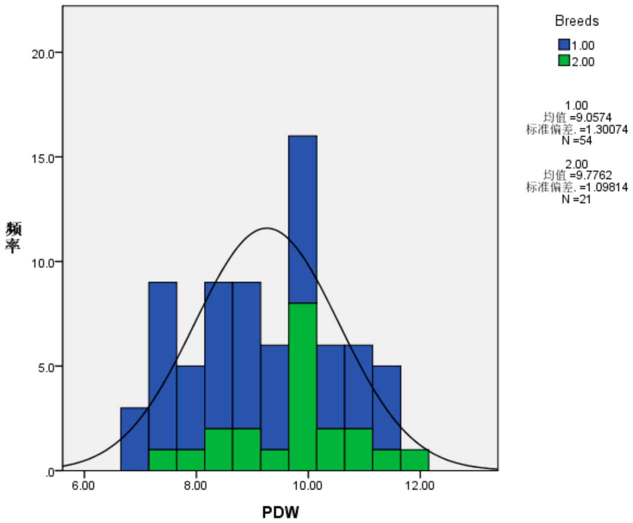
Breeds
1.00
2.00
1.00
均值 =9.0574
标准偏差. =1.30074
N =54
2.00
均值 =9.7762
标准偏差. =1.09814
N =21
频率
PDW

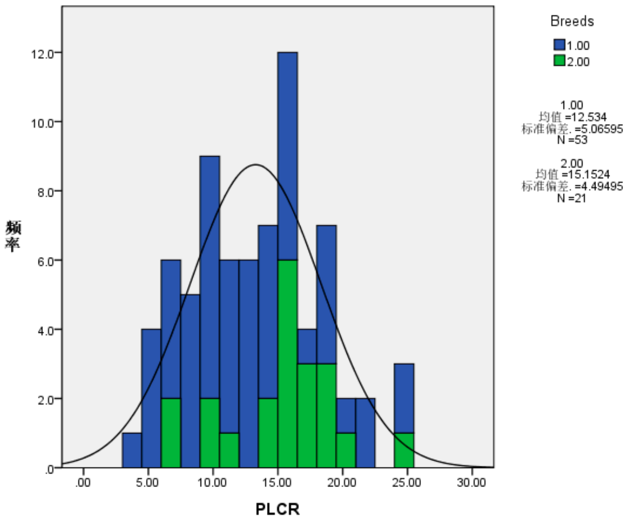
Breeds
1.00
2.00
1.00
均值 =12.534
标准偏差. =5.06595
N =53
2.00
均值 =15.1524
标准偏差. =4.49495
N =21
频率
PLCR

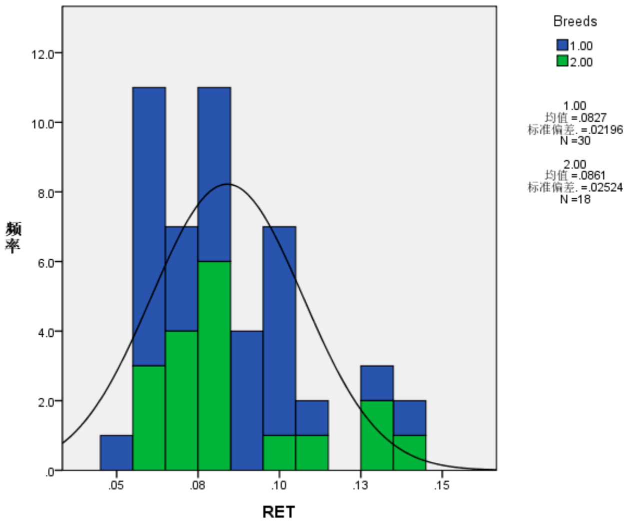
Breeds
1.00
2.00
1.00
均值 =.0827
标准偏差. =.02196
N =30
2.00
均值 =.0861
标准偏差. =.02524
N =18
频率
RET

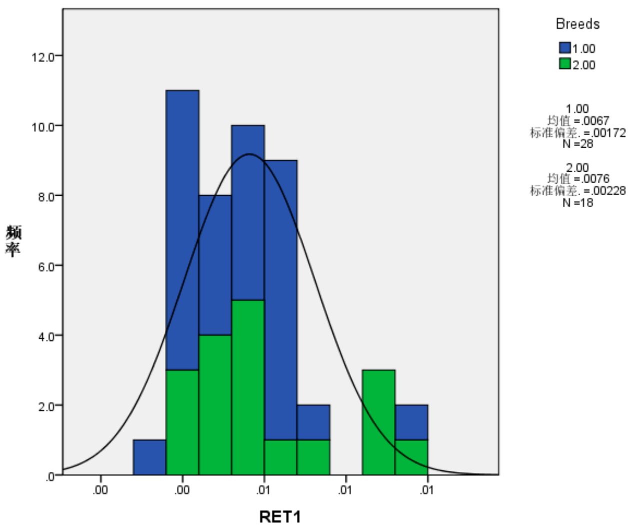

频率
12.0
10.0
8.0
6.0
4.0
2.0
.0
.00
.00
.01
.01
.01
RET1
Breeds
1.00
2.00
1.00
均值 =.0067
标准偏差. =.00172
N =28
2.00
均值 =.0076
标准偏差. =.00228
N =18
